# Do knowledge, knowledge sources and reasoning skills affect the accuracy of nursing diagnoses? a randomised study

**DOI:** 10.1186/1472-6955-11-11

**Published:** 2012-08-01

**Authors:** Wolter Paans, Walter Sermeus, Roos MB Nieweg, Wim P Krijnen, Cees P van der Schans

**Affiliations:** 1 Research and Innovation Group in Health Care and Nursing, Hanze University of Applied Sciences, post-box 3109, 9701 DC, Groningen, the Netherlands; 2 School of Public Health, Faculty of Medicine, Centre for Health Services and Nursing Research, Catholic University Leuven, Leuven, Belgium

**Keywords:** Clinical practice, Critical reasoning, Knowledge, Nursing diagnoses, RCT

## Abstract

**Background:**

This paper reports a study about the effect of knowledge sources, such as handbooks, an assessment format and a predefined record structure for diagnostic documentation, as well as the influence of knowledge, disposition toward critical thinking and reasoning skills, on the accuracy of nursing diagnoses.

Knowledge sources can support nurses in deriving diagnoses. A nurse’s disposition toward critical thinking and reasoning skills is also thought to influence the accuracy of his or her nursing diagnoses.

**Method:**

A randomised factorial design was used in 2008–2009 to determine the effect of knowledge sources. We used the following instruments to assess the influence of ready knowledge, disposition, and reasoning skills on the accuracy of diagnoses: (1) a knowledge inventory, (2) the California Critical Thinking Disposition Inventory, and (3) the Health Science Reasoning Test. Nurses (n = 249) were randomly assigned to one of four factorial groups, and were instructed to derive diagnoses based on an assessment interview with a simulated patient/actor.

**Results:**

The use of a predefined record structure resulted in a significantly higher accuracy of nursing diagnoses. A regression analysis reveals that almost half of the variance in the accuracy of diagnoses is explained by the use of a predefined record structure, a nurse’s age and the reasoning skills of `deduction’ and `analysis’.

**Conclusions:**

Improving nurses’ dispositions toward critical thinking and reasoning skills, and the use of a predefined record structure, improves accuracy of nursing diagnoses.

## Background

Nurses constantly make knowledge and skill-based decisions on how to manage patients’ responses to illness and treatment. Their diagnoses should be founded on the ability to analyse and synthesize patients’ information. Accurate formulation of nursing diagnoses is essential, since nursing diagnoses guide intervention [1-4]. It is part of a nurse’ professional role to verify his or her diagnosis with the patient, ‘to be sure that, in the patient’s judgement, the cue cluster represents a problem’ [5]. As stated by the World Alliance for Patient Safety [6], the lack of standardised nomenclature for reporting hampers good written documentation and may have a negative effect on patient safety internationally [7]. Based on a comparison of four classification systems, Müller-Staub [8] concluded that the NANDA-I classification is the best-researched and internationally most widely implemented classification system. The definition of ‘nursing diagnosis’ is: “A clinical judgment about individual, family or community responses to actual and potential health problems/life processes. A nursing diagnosis provides the basis for selection of nursing interventions to achieve outcomes for which the nurse is accountable” [9]. An accurate diagnosis describes a patient’s problem (label), related factors (aetiology), and defining characteristics (signs and symptoms) in unequivocal, clear language [1,3]. Describing a problem solely in terms of its label, in the absence of related factors and defining characteristics, can lead to misinterpretation [1,5,10]. Imprecise wording, lack of scrutiny, and expression of patient problems in terms of an incomprehensible diagnosis can have an undesirable effect on the quality of patient care and patient well-being [11,12]. Nevertheless, several authors have reported that patient records contain relatively few precisely formulated diagnoses, related factors, pertinent signs and symptoms, and poorly documented details of interventions and outcomes [13-15].

Numerous aspects related to cognitive capabilities and knowledge influence diagnostic processes [16,17]. Knowledge about a patient’s history and about how to interpret relevant patient information is a central factor in deriving accurate diagnoses [18-20]. A distinction between ‘ready knowledge’ and ‘knowledge obtained through the use of knowledge sources’ can be made. Ready knowledge is previously acquired knowledge that an individual can recall. Ready knowledge is achieved through education programmes and experience in situations in different nursing contexts [21,22]. Knowledge obtained through knowledge sources is acquired through the use of handbooks, protocols, pre-structured data sets, assessment formats, pre-structured record forms, and clinical pathways.

Knowledge sources may help nurses derive diagnoses that are more accurate than those derived without the use of such resources [23,24]. The purpose of using assessment formats based on Functional Health Patterns and standard nursing diagnoses, as included in the Handbook of Nursing Diagnoses [25,26], is to attain higher accuracy in diagnoses.

Another factor influencing the accuracy of nursing diagnoses is a nurse’ disposition towards critical thinking and reasoning skills. A disposition towards critical thinking includes open-mindedness, truth-seeking, analyticity, systematicity, inquisitiveness, and maturity [27]. Reasoning skills comprise induction and deduction, as well as analysis, inference, and evaluation. These skills are vital for the diagnostic process [27,28].

There are two kinds of diagnostic arguments: deductive and inductive. In a deductive argument the premises (foundation, idea, or hypothesis) supply complete evidence for the conclusion so that the conclusion necessarily follows from the premises. In an inductive (diagnostic) argument the premises provide some evidence, but are not completely informative with respect to the truth of the conclusion [29]. It could be noted, identification of a diagnosis is really only one aspect of the clinical reasoning process nurses engage. Clinical reasoning also involves some abduction related to diagnoses as well as interventions and outcomes.

Although, several studies have focused on nurses’ dispositions for critical thinking, these yielded little information about the influence of nurses’ specific reasoning skills in attaining high levels of accuracy of nursing diagnoses [28,30].

## Methods

### Aim

The aim of the study was twofold: (1) to determine whether the use of handbooks in nursing diagnoses, an assessment format subdivided in eleven health patterns and a predefined record structure subdivided in three sections ((1) problem label or diagnostic label, (2) aetiology of the problem and/or related factors and (3) signs/symptoms), affects the accuracy of nursing diagnoses; (2) to determine whether knowledge, disposition towards critical thinking, or reasoning skills influence the accuracy of nursing diagnoses.

### Design

A randomised, factorial design was used in 2009 to determine whether knowledge sources and a predefined record structure affect the accuracy of nursing diagnoses. Possible determinants—knowledge, disposition towards critical thinking, and reasoning skills—that could influence the accuracy of nursing diagnoses were measured by the following questionnaires: (1) a knowledge inventory; (2) the California Critical Thinking Disposition Inventory (CCTDI), a questionnaire that maps disposition towards critical thinking [31,32] and (3) the Health Science Reasoning Test (HSRT) [27].

### Sample

Clinical nurses were invited to derive diagnoses based on an assessment interview with a simulation patient (a professional actor) using a standardized script. Participants were randomly allocated to one of four groups—group A, B, C, and D. Group A could use knowledge sources (an assessment format with Functional Health Patterns, standard nursing diagnoses (labels), and handbooks of nursing diagnoses), and free text format (blank paper). Group B could use a predefined record structure (hereafter referred to as the “PES-format”), without knowledge sources, and group C could use both knowledge sources and a predefined record structure. Group D used neither knowledge sources nor the predefined record structure; they acted as a control group.

The entire procedure—starting from preparing for the assessment interview to writing the diagnoses—was recorded both on film and audiotape. During the interviews, an observer noted whenever the simulation patient failed to adhere to the script.

Of all 94 medical centres (86 general hospitals and 8 university hospitals) in the Netherlands in 2007, we randomly selected 11 hospitals, using stratification by province. Five hospital directors declined to participate. To replace these, we requested six additional hospital directors from the same region to participate. Of these, only one director refused to cooperate. The hospital directors approached the heads of nursing staff to request their participation. The heads of staff were asked to distribute registration forms to nurses to enrol in the study. We requested at least 20 registered nurses per hospital to participate.

### Data collection

First, the nurses were told that part of the study would be experimental, that they would be asked to complete questionnaires, and that the entire study would take about 2.5 hours of their time. None of the participants had any specific training in using NANDA-I diagnoses as a part of this study. We did not test whether the participating nurses already had knowledge of the NANDA classification or experience in the use of a PES-format.

Nurses’ allocation to each of the four groups took place by randomization i.e. by choosing one of four sealed envelopes. The form inside each envelope indicated allocation to group A, B, C, or D. The researchers were unaware of group allocation. Group A nurses (n = 49) were allowed to review the following knowledge sources in preparing for the assessment interview, and in formulating diagnoses:

1. An assessment format with Functional Health Patterns and standard nursing diagnoses (labels) as described in the Handbook of Nursing Diagnoses [26].

2. The Handbook of Nursing Diagnoses [26].

3. The Handbook of Nursing Diagnoses and NANDA-I classification (NANDA-I 2004) [9].

Seventy-nine nurses were allocated by randomisation to Group B. They did not have access to the aforementioned reference material. Instead, they had the opportunity to use a document with pre-structured sections to write down their diagnostic findings. One section of this document consisted of the ‘problem label’ (P, fill in…), the next section consisted of ‘related factors or aetiology’ (E, fill in…), and the last section consisted of ‘signs and symptoms’ (S, fill in…). Participants were required to state the problem label, related factors (aetiology) and the signs/symptoms or defining characteristics which corresponded with the patient’s condition. Based on the information in the script, presented by the actors, it was possible for nurses to complete information about P, E and S per diagnosis. The PES-format used by group B nurses listed one example of a nursing diagnosis noted in the PES-format and a brief introduction related to the example and ended with the following request: “Please note your diagnostic findings in the PES-format”. The example listed was not related to the case histories.

The fifty-one nurses in group C were allowed to review the knowledge sources along with the PES-format. They could take notes during the interview. Seventy nurses were randomly allocated to the control group D. Nurses in this group were given only a pen, notebook, and paper.

**Figure 1:**
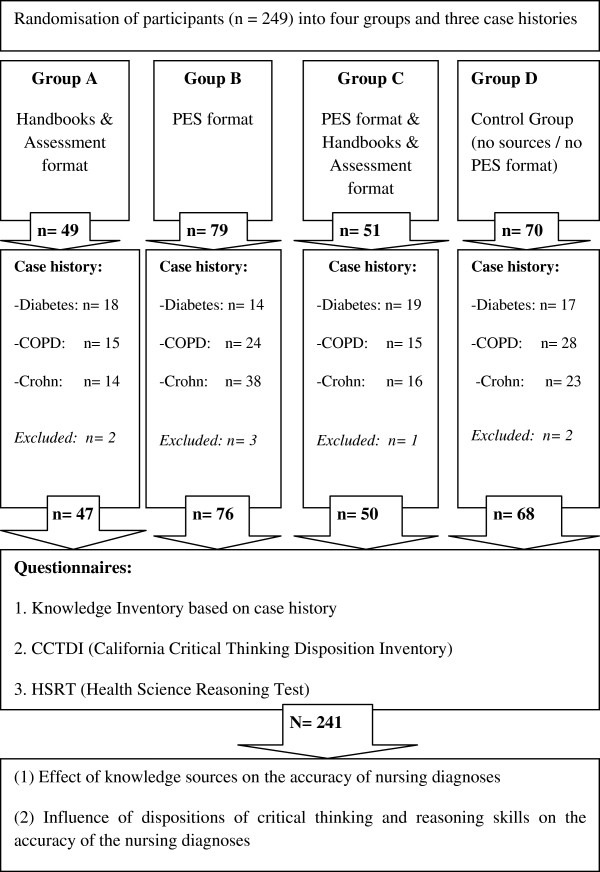
Research design.

For all groups, all items were within reach on the table, including pen, notebook, and paper. Each nurse was directed to an admission room and instructed to prepare for a 10 minute assessment interview with either a simulated diabetes mellitus type 1 patient (n = 71), a chronic obstructive pulmonary disease (COPD) patient (n = 84), or a Crohn’s disease patient (n = 93). Preparing for the assessment interviews, all nurses were given the following written information about the patient: name, gender, age and address; profession, family situation, and hobbies; medical history; the current medical diagnosis; reason for hospital admission and description of the current condition. Nurses were asked to derive accurate diagnoses based on the assessment interview and to note these on paper. Each participant assessed one professional actor representing a patient suffering from COPD, Crohn’s disease or diabetes mellitus type 1. Each of the three actors’ scripts contained six nursing diagnoses which should be identified by the nurses based on the assessment interview. Nurses in groups A and D documented their findings on blank paper. Nurses in groups B and C wrote their findings in the PES-format. After the interviews, the nurses were given 10 minutes to formulate their diagnoses. Based on video recordings, data from two control group nurses, two group A nurses, three group B nurses, and one group C nurse were excluded for the reason that the actors did not strictly adhere to the script in these cases. Finally, data from 241 nurses were analysed: 47 in group A, 76 in group B, 50 in group C and 68 in the control group, D. Of the 241 nurses, 68 assessed a diabetes patient, 82 assessed a COPD patient, and 91 assessed a patient with Crohn’s disease (Figure 1). The interviews lasted a maximum of 30 minutes (range: 10 – 30 minutes). Data collection of the questionnaires occurred directly after the experimental part of the study in a room nearby.

To ensure that participants could not prepare themselves or discuss any details of the study with others, all were asked to keep the contents and methods of the investigation confidential and to sign a corresponding agreement.

### Instrumentation

#### Case development

Three case scenarios were developed to include variety in the background of the simulation patients to mediate against knowledge and diagnostic documentation skills that were possibly related to knowledge of one specialty only. The development was based on the Guidelines for Development of Written Case Studies [3]. The cases were assessed by two external Delphi panels using a semi-structured questionnaire. The script was also assessed for clarity (clear language and sentence structure) and specificity, as well as nursing relevance. One of the Delphi panels consisted of lecturers with a master’s degree in nursing science (n = 6) and fourth-year bachelor’s degree nursing students (n = 2). The other Delphi panel consisted of experienced nurses working in clinical practice that had either a post-graduate specialization or a master’s degree in nursing science (n = 6). A physician screened the case scenarios for medical correctness. Any nursing diagnosis not belonging to the script was considered to be incorrect.

Subsequently, lecturers and students questioned the simulation patients during testing rounds, after which their answers were assessed for script consistency. All four actors involved had over five years of professional experience as simulation patients in nursing or medical education. Their acting was attuned to behaviour that was in accordance with the contents of the script. They were instructed to act like introverted, adequately responding patients to questions posed. The testing rounds were recorded both on film and tape and analysed by two lecturers and two bachelor’s students for script consistency and behaviour during the interviews.

After the last practice rounds, the script was considered to be fully consistent and was adopted for the study.

For measuring the accuracy of the nursing diagnoses, the D-Catch was used; an instrument that quantifies the degree of accuracy in written diagnoses [33,34]. This instrument consists of two sections: (1) Quantity, which addresses: “Are the components of the diagnosis present?” (scale: 1–4) and (2) Quality, which addresses: “What is the quality of the description with respect to relevancy, unambiguity, and linguistic correctness?” (scale: 1–4). For each diagnosis (out of six available) documented by each nurse, the sum of the quantity and quality criteria score was computed. From the six possible sum scores, the mean was taken and will be referred to as diagnoses accuracy score. Interrater reliability scores were computed from the quality and quantity scores.

The number of relevant diagnoses documented for each participant was determined out of six in total in each script. To be able to analyse whether nurses derived relevant diagnoses (accurate content), each of the three actors’ scripts contained six actual nursing diagnoses which should have been identified based on the assessment interview (Table 1). The scripts were specifically designed to represent exactly six actual nursing diagnoses. Any actual nursing diagnosis not fitting the script was considered to be irrelevant. Independent raters (n = 8; four registered nurses and four fourth year bachelor students in nursing) scored in pairs whether the diagnosis was considered to be relevant or not based on the listed six diagnoses after they had received 20 hours of training in reviewing the accuracy and relevancy of nursing diagnoses. Based on a consensus discussion, if necessary, raters gave a definite score. In almost all cases, the raters’ scores of relevant or irrelevant diagnosis did not lead to a discussion and the scores were found to be equal, because it was apparent whether a diagnosis was relevant and related to the six actual diagnoses in the script. Therefore no scores for Inter-rater reliability calculation were noted in the case of the six diagnoses from the fixed scripts.

**Table 1:** Nursing diagnoses in actors’ script

** Diagnostic labels**^**1**^**incorporated in the case history and script Diabetes patient**	**Diagnostic labels**^**1**^**incorporated in the case history and script COPD patient**	**Diagnostic labels**^**1**^**incorporated in the case history and script Crohn’s disease patient**
1 Fatigue (p. 253)	Activity intolerance (p. 66)	Chronic pain (p. 145)
2 Grieving (p. 280)	Anxiety (death anxiety) or fear (p. 72/p. 260)	Diarrhea (p. 225)
3 Impaired tissue or skin integrity (p. 464/p. 471)	Deficient fluid volume (p. 266)	Fatigue (p. 253)
4 Inactive self-health management (p. 579)	Disturbed sleep pattern (p. 602)	Ineffective activity planning (p. 71)
5 Ineffective activity planning (p. 71)	Impaired gas exchange (p. 516)	Ineffective coping (p. 199)
6 (Risk for) unstable blood glucose (p. 88)	Ineffective airway clearance (p. 511)	Stress overload (p. 647)

^1^ Diagnostic labels as published in: Nursing Diagnosis, Application to Clinical Practice, Edition 13, Carpenito-Moyet (2010), Lippincott Williams & Wilkins. [35].

#### Ready knowledge

A knowledge inventory was used only to determine the association between case-based conceptual, ready knowledge and the accuracy of nursing diagnoses. The knowledge inventory comprised of four case-related multiple-choice questions each consisting of four alternatives; with one correct answer. The Handbook of Enquiry & Problem Based Learning [36] was used as a guideline for development. The questions focused on content of the case presented. After assessors’ agreement was reached, the questions were adopted for the inventory.

#### Disposition towards critical thinking

Insight Assessment [37] was used to assess the influence of disposition towards critical thinking on the accuracy of nursing diagnoses. The CCTDI consists of 75 statements and measures respondents’ attitudes towards the use of knowledge and their disposition towards critical thinking. On a six-point scale, respondents indicated the extent to which they (dis)agreed with a certain statement.

The CCTDI consists of seven domains:

1. Truth-seeking: being flexible in considering alternatives and opinions.

2. Open-mindedness: being tolerant of divergent views with sensitivity to the possibility of one’s own bias.

3. Analyticity: being alert to potentially problematic situations, anticipating certain results or consequences.

4. Systematicity: being orderly and focused, aiming to correctly map out the situation both in linear and in non-linear problem situations.

5. Self-confidence: to be trusted upon for making adequate judgments.

6. Inquisitiveness: wanting to be well informed as well as having a desire to know how things work and fit together.

7. Maturity: making reflective judgments in situations where problems cannot be properly structured.

The scores of the CCTDI scales range from 10 to 60. The score indicates the degree of which nurses have a disposition toward critical thinking. Facione (2002) [32] found scores ranging from 10 to 30 to indicate an increasingly negative disposition; scores ranging from 40 to 60 to indicate an increasingly positive disposition; scores between 30 and 40 to indicate ambivalence (i.e. expression of positive or negative disposition). The recommended cut-off score for each scale is 40. A score of less than 40 reveals weakness [32].

Various studies have demonstrated the CCTDI scales to have a sufficient degree of reliability and validity [28,38-42]. Reliability of the Dutch version of the CCTDI quantified by Cronbach’s alpha was 0.74 (n = 241).

#### Reasoning skills

We used the HSRT to measure reasoning skills. The HSRT consists of 33 questions and assesses the reasoning capacity of healthcare and nursing professionals [27]. The HSRT was selected because its contents are usable in a context that is easily recognizable for nurses. The HSRT covers the following domains:

1. Analysis:

a. understanding the significance of experiences, opinions, situations, procedures, and criteria;

b. understanding connections between statements, questions, descriptions or presented convictions, experiences, reasons, sources of information, and opinions that may lead to a conclusion.

2. Inference: ability to formulate assumptions and hypotheses and to evaluate the relevancy of the information.

3. Evaluation:

a. ability to assess the credibility of statements, opinions, experiences, convictions, and to be able to determine relationships;

b. ability to reflect on procedures and results, to judge them, and to be able to provide convincing arguments for such.

4. Induction: ability to arrive at a general rule, which is more or less probable on the basis of a finite number of observations.

5. Deduction: ability to refine the truth of a conclusion; for example, the correct nursing diagnosis is guaranteed by the reasoning.

The HSRT subscales consist of six items to provide a guide for test takers’ abilities in the measured areas of Analysis, Inference, and Evaluation. For each of these subscales, a score of 5 or 6 indicates strong reasoning skills; a score of ≤ 2 indicates weak reasoning skills; and a score of 3 or 4 indicates average reasoning skills. Deductive and inductive scales consist of 10 items. For each of these subscales, a score of 8, 9, or 10 indicates strong deductive and inductive skills; and scores from 0 to 3 indicate weak deductive and inductive skills [27].

The HSRT test manual “The Health Sciences Reasoning Test” [27] is based on the consensus definition of critical thinking that was developed in the Delphi study described in the Expert Consensus Statement [32].

Linguistic validation of the CCTDI and the HSRT was done by forward and backward translation by two independent translators. The final translation was assessed by a third translator and approved to be relevant in the Dutch nursing context by a panel of nursing scientists (n = 6). Based on our study, reliability of the Dutch version of the HSRT was 0.72, quantified by Cronbach’s alpha (n = 241).

### Ethical considerations

For ethical reasons, the participants were informed that all information would be used for research purposes only and that data would be anonimized. By using the registration form, distributed in wards, nurses could subscribe voluntarily for participation. It was guaranteed that participation was during work time close to the ward of the nurses. Each of the participating nurses (n = 249) gave informed consent. In the Netherlands this research is not under the Ethical Committee’s restrictions.

### Statistical analysis

Demographic data were calculated by using SPSS version 15.0 and summarised by group means and standard deviations, along with percentages. Insight Assessment/The California Academic Press are the distributors of the CCTDI and HSRT. The scale scores of the CCTDI and HSRT were computed by Insight Assessment.

For the following statistics we used R version 2.10.1 (R Development Core Team 2009) [43]. Inter-observer agreement of the D-Catch was estimated by Cohen’s quadratic weighted kappa, intra-class correlation coefficient and Pearson’s product moment correlation coefficients of the first diagnoses listed by all of the participants. Cohen’s kappa with quadratic weighting was used to measure the proportion of agreement greater than that expected by chance. The intra-class correlation coefficient based on analysis of variance of the ratings gives the proportion of variance attributable to the objects of measurement [44]. The main and interaction effects of knowledge sources and PES-format on the accuracy of nursing diagnoses were estimated by two-way analysis of variance (ANOVA). The association between accuracy of diagnoses and knowledge, (knowledge inventory) dispositions towards critical thinking (CCTDI) and reasoning skills (HSRT), was estimated by Kendall’s tau. To estimate the amount of explained variance for the accuracy of nursing diagnoses (depended variable) we used linear regression, taking as independent variables the CCTDI domains and the HSRT scales as the knowledge inventory, presence of PES-format, knowledge sources and age of the participants.

## Results

### Demographic data

Licensed practical nurses (n = 53), hospital-trained nurses (n = 120), and bachelor’s degree nurses (n = 68), all working as qualified, registered nurses were included in our study; 64 percent had over 10 years of nursing experience. Ninety-two percent of the nurses worked at least 50 percent of full-time employment. Their mean (SD) age was 38 (10) years, and 212 (88 %) were female.

### Internal validity and reliability

We did not find significant differences between the four simulation patients on the accuracy of nursing diagnoses using two-way analysis of variance (p = 0.679), nor on the number of relevant diagnoses (p = 0.196). No significant differences were found between the three cases concerning the accuracy of the nursing diagnoses (p = 0.083) and the number of relevant diagnoses (p = 0.739). No significant differences were found between the CCTDI scores, the HSRT scores, and groups A, B, C, and D. We found no significant differences in the pairs of reviewers (n = 5), in the accuracy of the nursing diagnoses (p = 0.156), or on the number of relevant diagnoses (p = 0.546). These results are in line with the random assignment of nurses to groups.

Cohen’s weighted kappa, the intra-class correlation coefficient and Pearson’s product moment correlation coefficient, as well as their 95 percent confidence intervals are presented in Table 2. All of the coefficients are larger than .70 and have their left boundary of the confidence interval greater than .50.

**Table 2:** Inter-rater agreement measured Cohen´s Kappa with quadratic weighting, intra class correlation coefficient and Pearson’s product moment correlation coefficient with 95 % confidence intervals of quantity and quality criteria of the first diagnosis of each participant

**Raters**	**Objects N**^**a**^	***K*w**	**Intra Class Correlation**	**Pearson’s Correlation Coefficient**
Quantity				
1 vs 2	61	.82 (.70, .90)	.82 (.72, .89)	.82 (.72, .89)
3 vs 4	60	.83 (.75, .89)	.83 (.73, .89)	.84 (.74, .90)
2 vs 4	57	.72 (.55, .83)	.73 (.58, .83)	.73 (.57, .83)
5 vs 6	38	.82 (65,. 91)	.83 (.69, .91)	.82 (.68, .91)
7 vs 8	25	.75 (.61, .87)	.75 (.53, .88)	.75 (.53, .88)
Quality				
1 vs 2	61	.75 (.59, .85)	.75 (.62, .84)	.76 (.63, .85)
3 vs 4	60	.76 (.55, .83)	.76 (.63, .85)	.76 (.63, .85)
2 vs 4	57	.73 (.59, .84)	.73 (.59, .83)	.73 (.59, .84)
5 vs 6	38	.74 (.51,. 87)	.75 (.57, .86)	.74 (.56, .86)
7 vs 8	25	.79 (.55, .91)	.80 (.61, .90)	.80 (.60, . 90)

^a^Quality and quantity criteria of the accuracy measurement based on the D-Catch instrument.

### The influence of knowledge, handbooks/assessment format reasoning skills and the PES-format on the accuracy of diagnoses

In order to facilitate the interpretation of two-way ANOVA the means of the (in)depended variables over the experimental groups with PES-format and without PES-format are presented in Table 3. These means correspond to the main effects in two-way ANOVA, the significance of these are measured by the P-values. There is no significant main effect of PES-format or Handbooks/Assessment format on any of the CCTDI or HSRT domains on the number of relevant diagnoses. A significant PES-format effect was found on accuracy of nursing diagnoses (*F* = 118.5079, df = 1,237, p = < 0.0001). More specifically, the PES-format has an estimated increasing effect of 1.5 on mean diagnosis accuracy. There is no significant effect for Handbooks/Assessment format (*F* = 0.0786, df = 1,237, p = 0.7795) nor any significant interaction effects. The only exception to this is a significant interaction effect for Systematicity (CCTDI) but, neither its estimated size nor its corresponding main effects are significant. For these reasons we refrain from interpreting this effect.

**Table 3:** Group means (SD) and P-values from two-way ANOVA

**Scale**^**a**^	**Experimental conditions**						
**Groups**^**b**^	**A & D**	**B & C**	**B & D**	**A & C**	**Main effect PES-format P-Value**^**c**^	**Main effect Hand-Books & Assess-ment format P-Value**^**c**^	**Inter-action effect P-Value**^**c**^
**Dependent Variable**	**No PES-format**	**PES-Format**	**No Handbooks and no Assessment format**	**Handbooks & Assessment format**			
Accuracy of ND	4.0 (1.1)	5.4 (1.0)	4.8 (1.3)	4.7 (1.2)	<0.001*	0.780	0.956
Number of relevant ND	4.0 (1.3)	3.8 (1.2)	3.9 (1.3)	3.9 (1.3)	0.148	0.956	0.612
Knowledge Inventory	3.4 (1.1)	3.5 (1.2)	3.6 (1.2)	3.3 (1.2)	0.553	0.025*	0.936
CCTDI							
Truth-seeking	41.5 (5.9)	40.2 (5.5)	41.4 (4.9)	40.4 (6.1)	0.065	0.168	0.217
Open-Mindedness	37.9 (3.7)	37.3 (4.5)	37.2 (4.1)	37.8 (4.1)	0.303	0.308	0.268
Analyticity	43.5 (4.7)	44.2 (4.6)	44.4 (4.7)	43.5 (4.7)	0.259	0.174	0.067
Systematicity	44.8 (6.1)	46.2 (5.5)	45.9 (5.9)	45.3 (5.8)	0.063	0.441	0.039*
Self-confidence	44.2 (5.6)	44.2 (5.0)	44.8 (5.3)	43.9 (5.3)	0.953	0.186	0.207
Inquisitiveness	48.0 (5.8)	47.4 (5.3)	47.6 (6.0)	47.7 (5.2)	0.438	0.879	0.553
Maturity	44.0 (6.6)	44.3 (5.4)	44.3 (5.4)	44.0 (6.4)	0.693	0.754	0.173
HSRT							
Analysis	3.0 (1.4)	2.9 (1.2)	3.0 (1.4)	2.9 (1.2)	0.617	0.545	0.320
Inference	2.6 (1.1)	2.8 (1.4)	2.7 (1.2)	2.8 (1.3)	0.135	0.553	0.504
Evaluation	4.8 (1.1)	4.8 (1.0)	4.8 (1.1)	4.7 (1.0)	0.693	0.507	0.891
Induction	7.0 (1.4)	7.2 (1.5)	7.2 (1.5)	7.1 (1.4)	0.311	0.683	0.949
Deduction	4.6 (2.1)	4.7 (2.1)	4.8 (2.0)	4.6 (2.2)	0.521	0.373	0.760

^a^Mean (SD).

^b^Group A had the opportunity to use handbooks and assessment format and free text format (blank paper).

Group B had the opportunity to use a predefined record structure (PES-Format).

Group C had the opportunity to use handbooks and assessment format and a predefined record structure (PES-Format).

Group D did not have the opportunity to use handbooks and assessment format or PES-Format at all (control group).

^c^ ANOVA * P < .05.

Note: ND = nursing diagnoses; CCTDI = California Critical Thinking Disposition Inventory; HSRT = Health Science Reasoning Test.

Ready knowledge correlates with the main effect of handbooks and the assessment format because of higher mean scores as no handbooks or assessment format were available (p = 0.025, Table 3).

In order to estimate the degree of association between the dependent variable ‘Diagnoses Accuracy’ and the independent variables from the CCTDI and HSRT scales and the knowledge inventory, Kendall’s tau coefficients were computed. The resulting coefficients are presented in Table 4 together with the corresponding P-values.

**Table 4:** Kendall’s tau coefficients with corresponding P-values between mean diagnoses accuracy and the knowledge inventory, the CCTDI and the HSRT scales

**Variable**	**Kendall’s tau**	P-value*
Knowledge inventory	0.04	0.401
CCTDI		
Truth-seeking	0.01	0.756
Open-Mindedness	0.08	0.076
Analyticity	0.07	0.135
Systematicity	0.13	0.003*
Self-Confidence	0.07	0.121
Inquisitiveness	−0.01	0.899
Maturity	0.11	0.016*
HSRT		
Analysis	0.19	< 0.0001*
Inference	0.16	< 0.0001*
Evaluation	0.08	0.099
Induction	0.15	0.002*
Deduction	0.24	<0.0001*

*P < .05.

Note: CCTDI = California Critical Thinking Disposition.

Inventory; HSRT = Health Science Reasoning Test.

### Relationship of PES-format, handbooks/assessment format, age, CCTDI and HSRT scales, ready knowledge and diagnoses accuracy

By a regression analysis we investigated the degree to which variation in diagnostic accuracy can be explained by the following variables: age, the CCTDI and HSRT scales, as well as the knowledge inventory and the dichotomized variables PES-format and Handbooks/Assessment format (absence 0, presence 1). Using these (15) independent variables resulted in a multiple squared correlation of 0.4923, but also in a non-parsimonious model with several non-significant beta coefficients. To obtain a parsimonious linear regression model we used the stepwise approach according to Akaike’s information criterion and proceeded by manually dropping non-significant coefficients [45] -We merely note that doing this in different orders, resulted in one and the same model, reported in Table 5.- The resulting estimated linear model contains the dichotomized variable presence of PES-format, and the continuous variables of age and the reasoning skills of Deduction and Analysis (HSRT), see Table 5. Visual inspection of the fitted values by residuals as well as the normal quantile-quantile plot of residuals reveals that the latter is normally distributed with constant variance (normality is not rejected by the Shapiro-Wilk test). The model has a multiple squared correlation of 0.4702 and, therefore, explains 47.02 % of the variance in accuracy of the independent variable diagnoses. Almost half of the variance of diagnoses’ accuracy is explained by the presence of PES-format, nurse age and the reasoning skills of deduction and analysis. More specifically, the resulting model (Table 5) implies an increase of diagnostic accuracy by 1.37 if PES-format is present, a decrease of .025 if age increases by one year, an increase by .13 if deduction increases by one scale point, and an increase by .14 if analysis increases by one point. For a proper interpretation of these effects it is relevant to keep in mind the range of the (in)dependent variables. This is for diagnoses accuracy (2–8), for age (23–62), for deduction (0–10) and for analysis (0–6). Thus, according to the model, a nurse being 20 years younger has a larger mean accuracy of .5 (20 times 0.025). Similarly, an increase of deduction skills by 4 scale points yields an increase of mean diagnoses accuracy of 0.5 and an increase of analysis skills by 3 scale points yields an increase of mean accuracy by 0.43.

**Table 5:** **Model found by stepwise AIC**^**a**^followed by dropping non-significant coefficients*

**Model**	**Unstandardized Coefficients**		**t**	**P-Value**^**b**^
**Variables**	**B**	**SE**		
(Intercept)	4.0574	0.3202	12.67	≤ 0.0001
PES-format	1.3658	0.1213	11.26	≤ 0.0001
Age	−0.0251	0.0063	−4.00	≤ 0.0001
HSRT Deduction domain	0.1272	0.0347	3.66	0.0003
HSRT- Analysis domain	0.1442	0.0564	2.55	0.0113

* Dependent variable: accuracy of nursing diagnosis.

^a^AIC: Akaike's Information Criterion.

^b^P < .05.

Note: HSRT = Health Science Reasoning Test.

## Discussion

Because the PES-structure has been taught in nursing education programs in the Netherlands from the mid 1990’s onwards, this may explain why age was a significant predictor; younger nurses had higher accuracy scores than older nurses. Therefore we suggest that previous education in documenting in the PES-structure influences the accuracy of nursing diagnoses.

We did not find a significant main effect of using knowledge sources and the PES-format and the number of relevant diagnoses (Table 3). A possible explanation why PES did not affect the number of relevant diagnoses is that this format primarily guides the reasoning process of nurses, helping them to differentiate and document accurately.

### Ready knowledge

The finding that ready knowledge did not correlate with the accuracy of nursing diagnoses can be explained by the fact that the participants did not possess sufficient diagnostic skills needed to derive accurate diagnoses. In this study, knowledge of the problem area might have lead to more accurate diagnoses if the nurses had sufficient diagnostic skills to formulate these. Our findings are supported by Ronteltap (1990) [46] and Müller-Staub (2007) [15,47] who differentiated case-related knowledge from diagnostic skills as two essential aspects needed to report accurate diagnoses [21]. Ready knowledge did significantly correlate with the main effect of handbooks and the assessment format because of the higher scores recorded when no handbooks or assessment format were available. This significant finding was unexpected, since handbooks might be considered a knowledge stimulant and therefore knowledge looked up in handbooks might be thought to have a positive effect on the latter inventory scores. On the other hand, it is possible, in retrospect, that nurses are more activated to use ready knowledge in the absence of handbooks and the assessment format. It is not known whether this explanation is correct, or if our finding is coincidental, caused by multiple testing.

### Disposition towards critical thinking

Based on our findings, we assume nurses may need to be more aware of the importance of being sensitive to one’s potential bias and being alert to potentially problematic situations [48]. Nurse educators may need to teach students to reflect on disposition, to be orderly and focused, to aim correctly to map out situations, and to look in more depth for relevant diagnostic information. Several studies have reported that nurses trained in analytical skills score relatively highly on the CCTDI [41,49-53]. Therefore, nurses as well as lecturers may have to focus more on the development of positive thinking dispositions in daily hospital practice and in diagnostic training programs [54,55].

### Reasoning skills

Nurses with strong analysis, inference and deductive scores did stand out in the accuracy of their diagnoses. Previous studies in physician [56] and physical therapists [46] suggest that a diagnosis based on the use of deductive skills is more accurate than a diagnosis based on knowledge and experience alone. The relatively high scores on the inductive domain of the HSRT suggest that the nature of nurses’ reasoning is, to a certain extent, inductive. To properly determine which previous empirical information is relevant for a given clinical situation, nurses may use their inductive skills as well as their deductive and analytical skills [57].

#### Implications for education and clinical practice

The findings of our study may be of importance for education as well as for clinical practice as they provide resources that positively influence nursing diagnosis documentation. Teaching nursing students and nurses in practice how to employ strategies to use ready knowledge and knowledge sources is an essential objective for nursing as it guides nurses to accuracy in nursing diagnosis documentation [12]. We provide evidence that nurses’ dispositions towards critical thinking and their diagnostic reasoning skills are vital to obtain accurate nursing diagnoses that serve as the basis for selection of interventions and the achievement of patient outcomes. This study gives evidence that the PES format increases accuracy in nursing diagnosis. We assume that the PES format may be useful in clinical practice for nurses, facilitators, administrators and record designers as they have their responsibility in providing accurate nursing documentation. We suggest that the use of the PES format should be incorporated into digital nursing documentation systems. These systems, including resources as pre-formulated templates, have positive influences on the frequency of diagnoses documentation and the time needed to obtain a diagnosis is significantly shorter in combination with a computer aid [58,59]. The use of knowledge resources to reduce the lack of precision of diagnostic reports may improve nurses’ documentation [22,28] and help them to overcome time-consuming reports with useless redundancies as was found recently in several studies in nursing documentation [33,34]. The evidence for the relationship between specific reasoning skills and accuracy in nursing diagnoses may contribute to the development of nursing education programs and assessments addressing these reasoning skills in several countries in which these skills have less attention. We conclude that case-related knowledge, critical thinking and reasoning skills need to be taught and assessed comprehensively in nursing schools and in post education programmes if nurses are to avoid inaccurate diagnoses and incorrect interventions in clinical practice.

#### Limitations

Nurses in our sample voluntarily participated in a study in diagnostics, even though they were uncertain of what to expect in the case history contents and questionnaires. This method of sampling may have introduced a selection bias, since a number of nurses in our sample might have been more focused on nursing diagnoses. We did not collect data on participants’ knowledge of NANDA-I classification or the PES format. Therefore we were not able to associate available knowledge related to these issues to the diagnoses’ accuracy scores; this can be seen as a limitation of the study. Using actors as simulation patients, we were able to create an experimental environment that closely resembled the participants’ work setting. However, actors are not the same as patients. We have to consider the possibility that the information presented by the actors had more internal coherence than information obtained from real patient situations, and therefore was easier to assess. On the other hand, working with actors provided us with natural nurse-to-patient interactions.

This study focuses on reasoning skills and critical thinking dispositions as defined by Facione [31,32] and addresses the accuracy of nursing diagnosis. We did not study nurses’ reasoning approach in selecting nursing interventions or outcome evaluations, because interpretations of patient data serve as the basis for selecting the nursing interventions that will achieve positive patient outcomes [3].

The literature suggests that experienced nurses do not centre on inductive and deductive diagnostic inference only in their decision making [47,48,60,61]. They are able to choose the most likely hypothesis to explain their observations and to adopt this hypothesis as a starting point of further analysis. This is a process, -known as ‘abductive reasoning’-, of choosing a hypothesis, which would best explain the available evidence [21]. ‘Abduction is the first stage of inquiry within which hypotheses are invented; they are then explicated through deduction and verified through induction’ [62]. This reasoning approach was not considered in this study. Future research may allow more insight in how abductive, inductive and deductive reasoning influences accuracy and relevancy in nursing diagnosis, interventions and outcomes.

#### Advance on previous publication

A previous publication in the Journal of Professional Nursing [61] is related to this study. This was a pilot study in a randomized controlled trial design in two groups that examined our methodological approach to studying how nursing students (n = 100) derive diagnoses. Our approach proves to be feasible. Therefore, this study entitled: ‘Do knowledge, knowledge sources and reasoning skills affect the accuracy of nursing diagnoses?’ can be seen as a more comprehensive follow up study of registered nurses.

## Conclusion

Based on the results of this study it can be concluded that the PES-format has a main positive effect on the accuracy of the nursing diagnoses. Almost half of the variance of diagnoses accuracy is explained by the presence of PES-format, nurse age and the reasoning skills of deduction and analysis. As far as we know this result is new and gives a new perspective to pre-structured formats and reasoning skills. More analytical and deductive reasoning needs to be taught to accurately use the PES-structure in nursing education programs and in computer software in electronic record systems. Part of the 53 % unexplained variance is likely to be random in the sense that it cannot be explained by systematic variance from independed variables. Personal knowledge, experience, and (subjective) individual reflections are part of nurses’ diagnostic process as well [3,4,60,63,64].

The results of this study should have implications for nursing practice and education. Improving nurses’ disposition towards critical thinking, improving nurses’ reasoning skills, and encouraging them to use the PES-structure, could be a step forward in improving the accuracy of nursing diagnoses.

## Competing interests

The authors declare that they have no competing interests.

## Author contributions

WP, WS & CvdS were responsible for the study conception and design, WP performed the data collection. WP, WS, WK & CvdS performed the data analysis, WP, WS, RN & CvdS were responsible for the drafting of the manuscript, WP, WS, RN, WK & CvdS made critical revisions to the paper for important intellectual content, WP, WS,WK & CvdS provided statistical expertise, WP, WS, RN & CvdS provided administrative, technical and material support, WS, RN & CvdS supervised the study. All Authors read and approved the final manuscript.

## Pre-publication history

The pre-publication history for this paper can be accessed here:

http://www.biomedcentral.com/1472-6955/11/11/prepub

## References

[B1] Gordon M Nursing diagnoses process and application 1994 McGraw Publishers, New York

[B2] Gordon M Clark J Naming Nursing Capturing Patient Problems: Nursing Diagnosis and Functional Health Patterns 2003 Hans Huber, Bern 87 94

[B3] Lunney M Critical Thinking and Nursing Diagnoses 2001 Case Studies and Analyses, North American Nursing Diagnosis Association

[B4] Lunney M Current knowledge related to intelligence and thinking with implications for the development and use of case studies Int J Nurs Terminol Classif 2008 19 4 158 162 10.1111/j.1744-618X.2008.00104.x 19128334

[B5] Gordon M Implementing nursing diagnoses, interventions and outcomes – Functional health patterns: assessment and nursing diagnosis 2005 ACENDIO. Hans Huber, Bern 58 59

[B6] World Alliance for Patient Safety World Health Organization, W.H.O. Guidelines for Safe Surgery 2008 First WHO Press, Geneva 127 128

[B7] Zeegers HWM Adverse events among hospitalized patients. Results and Methodological aspects of a record review study 2009 PHD thesis, Vrije Universiteit Amsterdam

[B8] Müller-Staub M Evaluation of the implementation of nursing diagnostics; A study on the use of nursing diagnoses, interventions and outcomes in nursing documentation 2007 PhD-thesis, Elsevier/Blackwell Publishing, Print, Wageningen

[B9] NANDA International Handbook Nursing Diagnoses: Definitions & Classification 2004 2004 NANDA International, Philadelphia

[B10] Lunney M Critical Thinking and Accuracy of Nurses’ Diagnoses Int J Nurs Terminol Classif 2003 14 3 96 107 10.1111/j.1744-618X.2003.tb00068.x 14649031

[B11] Kautz DD Kuiper RA Pesut DJ Williams RL Using NANDA, NIC, NOC (NNN) Language for Clinical Reasoning With the Outcome-Present State-Test (OPT) Model Int J Nurs Terminol Classif 2006 17 3 129 138 10.1111/j.1744-618X.2006.00033.x 17117929

[B12] Lunney M The critical need for accuracy of diagnosing human responses to achieve patient safety and quality-based services 2007 ACENDIO Oud Consultancy, Amsterdam 238 239

[B13] Ehrenberg A Ehnfors M Thorell-Ekstrand I Nursing documentation in patient records: experience of the use of the VIPS model J Adv Nurs 1996 24 853 867 10.1046/j.1365-2648.1996.26325.x 8894904

[B14] Moloney R Maggs C A systematic review of the relationship between written and manual nursing care planning, record keeping and patient outcomes J Adv Nurs 1999 30 51 56 10.1046/j.1365-2648.1999.01048.x 10403980

[B15] Müller-Staub M Lavin MA Needham I van Achterberg T Nursing Diagnoses, Interventions and Outcomes- Application and Impact on Nursing Practice: Systematic Literature Review J Adv Nurs 2006 56 5 514 531 10.1111/j.1365-2648.2006.04012.x 17078827

[B16] Smith M Higgs J Ellis E Higgs JJ MA, Loftus S, Christensen N Clinical reasoning in the health professions Factors influencing clinical decision making 2008 third Elsevier, Amsterdam

[B17] Reed PG Lawrence CA A paradigm for the production of practice-based knowledge J Nurs Manag 2008 16 4 422 432 10.1111/j.1365-2834.2008.00862.x 18405259

[B18] Cholowski KM Chan LKS Diagnostic reasoning among second year nursing students J Adv Nurs 1992 17 1171 1181 10.1111/j.1365-2648.1992.tb01832.x 1430619

[B19] Hasegawa T Ogasawara C Katz EC Measuring Diagnostic Competency and the Analysis of Factors Influencing Competency Using Written Case Studies Int J Nurs Terminol Classif 2007 18 3 93 102 10.1111/j.1744-618X.2007.00057.x 17714237

[B20] Lee J Chan ACM Phillips DR Diagnostic practice in nursing: A critical review of the literature Nurs Heal Sci 2006 8 57 65 10.1111/j.1442-2018.2006.00267.x 16451430

[B21] Gulmans J Leren Diagnosticeren, Begripsvorming en probleemoplossen in (para)medische opleidingen 1994 Thesis Publishers, Amsterdam

[B22] Lunney M Helping Nurses Use NANDA, NOC and NIC, Novice to Expert J Nurs Adm 2006 36 3 118 125 10.1097/00005110-200603000-00004 16601513

[B23] Goossen WTF Towards strategic use of nursing information in the Netherlands, Northern Centre for Healthcare Research 2000 PhD-thesis, Groningen Academic Publishers

[B24] Spenceley SM O’ Leavy KA Chizawski LL Ross AJ Estabrooks CA Sources of information used by nurses to inform practice: An integrative review Int J Nurs Stud 2008 45 6 945 970 10.1016/j.ijnurstu.2007.06.003 18677809

[B25] Carpenito LJ Johnnson CRM The NANDA definition of nursing diagnosis Classifications of Nursing Diagnoses, Proceedings of the Ninth Conference 1991 J.B. Lippincott Company, Philadelphia

[B26] Carpenito LJ Zakboek verpleegkundige diagnosen 2002 Wolters-Noordhoff, Groningen

[B27] Facione NC Facione PA The Health Sciences Reasoning Test 2006 The California Academic Press, Milbrae, CA

[B28] Stewart S Dempsey LF A Longitudinal study of baccalaureate nursing students’ critical thinking dispositions J Nurs Educ 2005 44 2 65 70 15719715 10.3928/01484834-20050201-07

[B29] Bandman EL Bandman B Critical thinking in nursing 1995 2 Appleton & Lange, Norwalk, Connecticut

[B30] Tanner CA What have we learned about critical thinking in nursing? J Nurs Educ 2005 44 2 47 48 15719709 10.3928/01484834-20050201-01

[B31] Facione NC Facione PA Sanchez CA Critical thinking disposition as a measure of competent clinical judgment: The development of the California Critical Thinking Disposition Inventory J Nurs Educ 1994 33 8 345 350 7799093 10.3928/0148-4834-19941001-05

[B32] Facione PA Critical Thinking: A Statement of Expert Consensus for Purposes of Educational Assessment and Instruction, Executive Summary: The Delphi Report, 2000 2001 The California Academic Press, Milbrae, CA

[B33] Paans W Sermeus W Nieweg MB van der Schans CP D-Catch instrument: development and psychometric testing of a measurement instrument for nursing documentation in hospitals J Adv Nurs 2010 66 6 1388 1400 10.1111/j.1365-2648.2010.05302.x 20546369

[B34] Paans W Sermeus W Nieweg MB van der Schans CP Prevalence of accurate nursing documentation in the patient record in Dutch hospitals J Adv Nurs 2010 66 11 2481 2490 10.1111/j.1365-2648.2010.05433.x 20735494

[B35] Carpenito LJ Diagnostic labels Nursing Diagnosis, Application to Clinical Practice 2010 13 Lippincott Williams & Wilkins, Philadelphia

[B36] Barrett T, Mac Labhrainn I, Fallon H Handbook of Enquiry & Problem Based Learning 2005 Creative Commons, Galway

[B37] Insight Assessment California Critical Thinking Disposition Inventory Test Manual, Score Interpretation Document 2002 The California Academic Press, Milbrae, CA

[B38] Kakai H Re-examining the factor structure of the California Critical Thinking Disposition Inventory J Perception on Motivational Skills 2003 96 2 435 438 10.2466/pms.2003.96.2.435 12776825

[B39] Kawashima A Petrini MA Study of critical thinking skills in nursing diagnoses in Japan Nurs Educ Today 2004 24 4 188 195 10.1016/j.nedt.2004.02.001 15110438

[B40] O’Neil ES Dluhy NM A Longitudinal framework for fostering critical thinking and diagnostic reasoning J Adv Nurs 1997 26 4 825 832 10.1046/j.1365-2648.1997.00338.x 9354998

[B41] Tiwari A Avery A Lai P Critical Thinking Disposition of Hong Kong Chinese and Australian Nursing Students J Adv Nurs 2003 44 3 298 307 10.1046/j.1365-2648.2003.02805.x 14641400

[B42] Yeh ML Assessing the reliability and validity of the Chinese version of the California Critical Thinking Disposition Inventory Int J Nurs Stud 2002 39 2 123 132 10.1016/S0020-7489(01)00019-0 11755443

[B43] R Development Core Team R A language and environment for statistical computing 2009 R Foundation for Statistical Computing, Vienna, Austria http://www.R-project.org

[B44] McGraw KO Wong SP Forming inferences about some intra-class correlation coefficients Psychological Methods 1996 1 1 30 46

[B45] Venables WN Ripley BD Statistics and Computing, Modern Applied Statistics with S 2002 Fourth Springer, New York

[B46] Ronteltap CFM De rol van kennis in fysiotherapeutische diagnostiek; psychometrische en cognitief-psychologische studies 1990 PHD-Thesis, Thesis Publishers Amsterdam

[B47] Müller-Staub M Needham I Obenbreit M Lavin A van TH A Improved Quality of Nursing Documentation: Results of a Nursing Diagnoses, Interventions, and Outcomes Implementation Study Int J Nurs Terminol Classif 2006 18 1 5 17 10.1111/j.1744-618X.2007.00043.x 17430533

[B48] le Fevre Alfaro R Critical thinking and clinical judgment 2004 third Saunders/Elsevier, Missouri 7 36

[B49] Colucciello ML Critical thinking skills and dispositions of baccalaureate nursing students: a conceptual model for evaluation J Prof Nurs 1997 13 4 236 245 10.1016/S8755-7223(97)80094-4 9239982

[B50] Wan YI Lee D Chau J Wootton Y Chang A Disposition towards critical thinking; A study of Chinese undergraduate nursing students J Adv Nurs 2000 32 84 89 10.1046/j.1365-2648.2000.01417.x 10886438

[B51] Profetto-McGrath J Hesketh KL Lang S Study of Critical Thinking and Research Among Nurses West J Nurs Res 2003 25 3 322 337 10.1177/0193945902250421 12705114

[B52] Profetto-McGrath J The relationship of critical thinking skills and critical thinking dispositions of baccalaureate nursing students J Adv Nurs 2003 43 6 569 577 10.1046/j.1365-2648.2003.02755.x 12950562

[B53] Nokes KM Nickitas DM Keida R Neville S Does Service-Learning Increase Cultural Competency, Critical Thinking, and Civic Engagement? J Nurs Educ 2005 44 2 65 70 15719713 10.3928/01484834-20050201-05

[B54] Björvell C Nursing Documentation in Clinical Practice, instrument development and evaluation of a comprehensive intervention programme 2002 PhD thesis, Karolinska Institutet, Stockholm

[B55] Cruz DM Pimenta CM Lunney M Improving critical thinking and clinical Reasoning with a continuing education course J Contin Educ Nurs 2009 40 3 121 127 10.3928/00220124-20090301-05 19326819

[B56] Barrows HS Norman GR Neufeld VR Feightner JW The clinical reasoning of randomly selected physicians in general practice Clin Invest Med 1982 5 49 55 7116714

[B57] Lee TT Nursing diagnoses: factors affecting their use in charting standardized care plans J Clin Nurs 2005 14 5 640 647 10.1111/j.1365-2702.2004.00909.x 15840079

[B58] Kurashima S Kobayashi K Toyabe S Akazawa K Accuracy and efficiency of computer aided nursing diagnosis Int J Nurs Terminol Classif 2008 19 3 95 101 10.1111/j.1744-618X.2008.00088.x 18798940

[B59] Gunningberg L Fogelberg-Dahm M Ehrenberg A Improved quality comprehensiveness in nursing documentation of pressure ulcers after implementing an electronic health record in hospital care J Clin Nurs 2009 18 11 1557 1564 10.1111/j.1365-2702.2008.02647.x 19220607

[B60] Pesut DJ Herman J Clinical Reasoning, The Art en Science of Critical and Creative Thinking 1999 Delmar Publishers, Washington

[B61] Paans W Sermeus W Nieweg MB van der Schans CP Determinants of the accuracy of nursing diagnoses: influence of ready knowledge, knowledge sources, disposition toward critical thinking, and reasoning skills J Prof Nurs 2010 26 4 232 241 10.1016/j.profnurs.2009.12.006 20637445

[B62] Råholm MB Abductive reasoning and the formation of scientific knowledge within nursing research Nurs Philos 2010 11 4 260 10.1111/j.1466-769X.2010.00457.x 20840137

[B63] Benner P Tanner C Clinical judgment: how expert nurses use intuition Am J Nurs 1987 87 1 23 31 3642979

[B64] Cutler P Problem solving in clinical medicine. From data to diagnosis 1979 The Williams and Wilkins Company, Baltimore

